# Anthropometric Indicators as a Tool for Diagnosis of Obesity and Other Health Risk Factors: A Literature Review

**DOI:** 10.3389/fpsyg.2021.631179

**Published:** 2021-07-09

**Authors:** Paola Piqueras, Alfredo Ballester, Juan V. Durá-Gil, Sergio Martinez-Hervas, Josep Redón, José T. Real

**Affiliations:** ^1^Instituto de Biomecánica de Valencia, Universitat Politècnica de Valencia, Valencia, Spain; ^2^Service of Endocrinology and Nutrition, Hospital Clínico Universitario de Valencia, Valencia, Spain; ^3^Institute of Health Research of the Hospital Clinico Universitario de Valencia (INCLIVA), Valencia, Spain; ^4^Department of Medicine, University of Valencia, Valencia, Spain; ^5^CIBER de Diabetes y Enfermedades Metabólicas Asociadas (CIBERDEM), Madrid, Spain; ^6^Department of Internal Medicine, Hospital Clínico de Valencia, University of Valencia, Valencia, Spain; ^7^CIBER Fisiopatología Obesidad y Nutrición (CB06/03), Instituto de Salud Carlos III, Madrid, Spain; ^8^Cardiovascular and Renal Risk Research Group, Institute of Health Research of the Hospital Clinico Universitario de Valencia (INCLIVA), University of Valencia, Valencia, Spain

**Keywords:** obesity, anthropometric health indicators, health, risk identification, fat distribution, 3D human shapes

## Abstract

Obesity is characterized by the accumulation of an excessive amount of fat mass (FM) in the adipose tissue, subcutaneous, or inside certain organs. The risk does not lie so much in the amount of fat accumulated as in its distribution. Abdominal obesity (central or visceral) is an important risk factor for cardiovascular diseases, diabetes, and cancer, having an important role in the so-called metabolic syndrome. Therefore, it is necessary to prevent, detect, and appropriately treat obesity. The diagnosis is based on anthropometric indices that have been associated with adiposity and its distribution. Indices themselves, or a combination of some of them, conform to a big picture with different values to establish risk. Anthropometric indices can be used for risk identification, intervention, or impact evaluation on nutritional status or health; therefore, they will be called anthropometric health indicators (AHIs). We have found 17 AHIs that can be obtained or estimated from 3D human shapes, being a noninvasive alternative compared to X-ray-based systems, and more accessible than high-cost equipment. A literature review has been conducted to analyze the following information for each indicator: definition; main calculation or obtaining methods used; health aspects associated with the indicator (among others, obesity, metabolic syndrome, or diabetes); criteria to classify the population by means of percentiles or cutoff points, and based on variables such as sex, age, ethnicity, or geographic area, and limitations.

## Introduction

Overweight and obesity are the most prevalent metabolic disorders in developed countries. The prevalence of obesity has increased tremendously in recent decades (WHO | Noncommunicable diseases country profiles, 2018). In Spain, the estimated prevalence of overweight [body mass index (BMI), 25.0–29.9] in the adult population reaches 39.3%, and the global prevalence of obesity (BMI> 30) is estimated at 21.6% (Aranceta-Bartrina et al., 2016).

Obesity is characterized by abnormal or excessive fat accumulation. Obesity has been associated with an increased risk of type 2 diabetes and cardiovascular disease, as well as other conditions such as cancer, mental health, and osteoarthritis, contributing to a decrease in both quality of life and life expectancy (Pischon et al., 2008; Di Angelantonio et al., 2016; Blüher, 2019).

The obese phenotype, however, is complex, and some patients have no obvious cardiometabolic effect. In this sense, obesity, but especially abdominal adiposity (central or visceral), induces or aggravates the presence of insulin resistance, which, in turn, leads to different metabolic disturbances, constituting a cluster of the obesity-driven alterations also known as the metabolic syndrome (Kahn et al., 2019).

Taking into account that obesity is a known independent risk factor for non-communicable diseases and that the increasing prevalence of obesity worldwide confers a significant global public health burden (Bray et al., 2016), it is necessary to prevent, detect, and appropriately treat obesity to reduce the future health and economic costs of this problem. The first step to achieve this is to reliably diagnose individuals. In this sense, many anthropometric indices associated with adiposity and its distribution have emerged, such as the widely known BMI, waist-to-hip ratio (WHR), or body fat percentage (BFP).

Anthropometric indices have been used for different purposes becoming indicators for risk identification, intervention, or impact assessment on nutritional status or health. Therefore, from now on, these indices will be called anthropometric health indicators (AHIs). There are many studies related to AHIs in the literature in which estimation methods are proposed, cutoffs are provided for different populations, or in which their reliability and validity are analyzed. However, its impact as a whole has not been fully established and analyzed. The aim of this review is to provide an overview of different AHIs and to review in depth those that can be obtained or estimated from 3D human shapes and their usefulness for assessing obesity and other health risk factors. These AHIs are on the rise, especially with the potential of emerging 3D imaging devices, compared to traditional laboratory methods, which are invasive, require well-trained laboratory personnel, and are often too expensive for field settings.

## Methods

### Search Criteria

A total of 42 AHIs have been previously identified. In order to categorize them, two questions were raised:

- *Can the AHI be obtained or estimated from 3D human shapes?*
- *Does the AHI have enough available classification criteria? Can the AHI be used to assess health risk of individuals even if limited?*

**Figure 1:**
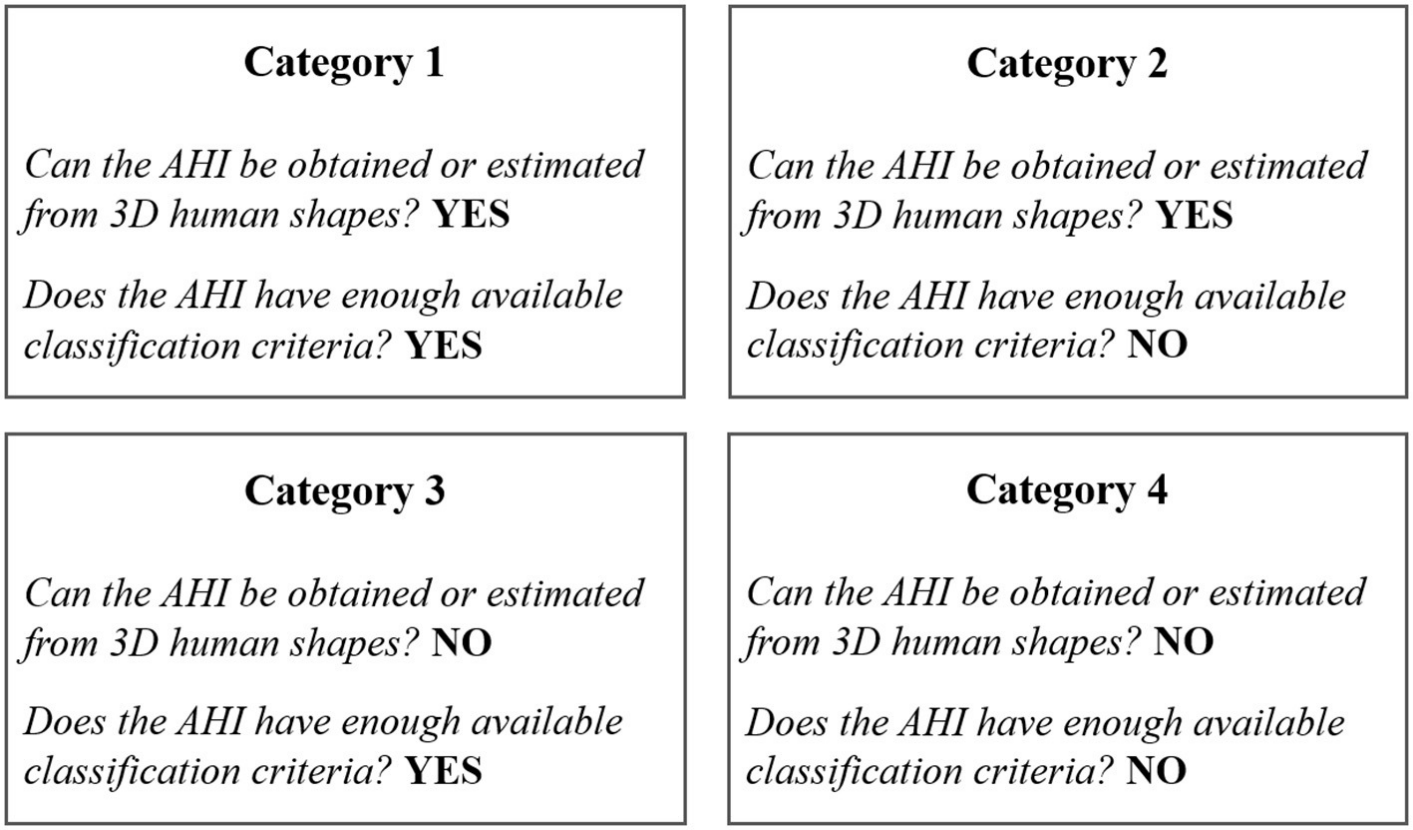
Classification of AHIs into four categories.

Based on the answers to these two questions, the AHIs have been grouped into four categories (Figure 1). A list of the AHIs grouped into the four categories is shown in Table 1.

**Table 1:** List of AHIs grouped into the four categories.

**Category 1**
Body Mass Index (BMI)
Waist circumference (WC)
Waist-to-Hip Ratio (WHR)
Waist-to-Height Ratio (WHtR)
Body Fat Percentage (BFP)
Conicity Index (CI)
Sagittal Abdominal Diameter (SAD)
Abdominal Volume Index (AVI)
Visceral Adipose Tissue Area (VATA)
Fat Free Mass Index (FFMI)
Fat Mass Index (FMI)
Primary Shape and Shape Tendency (PS&ST)
Trunk Fat (TF)
Trunk Fat Percentage (TFP)
A Body Shape Index (ABSI)
Body Roundness Index (BRI)
Neck circumference (NC)
**Category 2**
Fat Mass (Total Body Fat) (FM)
Trunk to Leg Volume Ratio (TLVR)
Thigh to Abdomen-Hip Volume Ratio (THVR)
Body Volume Index (BVI)
Subcutaneous Adipose Tissue Area (SATA)
Transverse Abdominal Diameter (TAD)
Sagittal-Transverse Abdominal Diameter Ratio (STR)
Face Morphology (FMO)
Average Density (AD)
Waist to Thigh Ratio (WTR)
Leg to Whole-Body Fat Mass Ratio (LBFR)
Leg to Trunk Fat Mass Ratio (LTFR)
Arm circumference (biceps) (AC)
Logarithmic Body Score Index Z-score (LBSIZ)
Body Mass Index z-scores (BMIz)
Percent of Ideal Body Weight (%IBW)
**Category 3**
Visceral Adiposity Index (VAI)
**Category 4**
Visceral and Subcutaneous Adipose “Depth” (VSAD)
Visceral/Subcutaneous Fat Ratio (VSR)
Subcutaneous Adipose Tissue Volume (SATV)
Visceral Adipose Abdominal Tissue (Volume) (VAAT)
Subcutaneous Adipose Abdominal Tissue (Volume or Mass) (SAAT)
Total Adipose Tissue Volume (TATV)
Leg Fat Mass Percentage (LFM%)
Somatotype

Methods of the analysis and inclusion criteria were specified in advance and documented in a protocol.

### Inclusion Criteria

The specific inclusion and exclusion criteria are shown in Table 2. Basically, criteria for the inclusion of studies included the following:

1. Studies published from January 1956 until September 30, 2020.
2. Studies performed in humans.
3. Studies published in English language.
4. Studies shown a direct anthropometric measurement, prediction equations, models, or methods based on equipment measurements such as bioelectrical impedance analysis (BIA) or dual-energy X-ray absorptiometry (DXA).
5. Studies provided the health aspects associated with the indicator, among others, obesity, metabolic syndrome, or diabetes.
6. Studies presented information on criteria to classify the population—by means of percentiles or cutoff points, and based on variables such as sex, age, ethnicity, or geographic area—and limitations.

**Table 2:** Inclusion and exclusion criteria according to PICO.

**Criteria**	**Inclusion**	**Exclusion**
Population	Human. Adults and/or children	Animal
Intervention	AHI obtained or estimated from 3D human shapes; AHI with availability of classification criteria	
Outcome	AHI's obtaining method; AHI's associated health aspect; AHI's classification criteria; AHI's limitation	
Comparison	Other method to assess body composition	
Date	Cutoff date limit of 1956–2020 was applied	
Language	Only studies written in English were included	
Field of search	Title or abstract	

### Literature Research

The literature search was performed in the electronic database of PubMed, including the 17 AHIs, which can be obtained or estimated from 3D human shapes (Table 1; Category 1).

The key words analyzed and the search strategy are shown in detail as follows: (diabetes OR metabolic syndrome OR cardiometabolic OR Cut-off OR cutoff OR cut off OR Blood pressure OR Classification OR obesity OR cardiovascular disease OR fat mass OR visceral fat OR fat distribution OR mortality OR children OR adolescent OR gender OR formula OR bioelectrical impedance accuracy OR anthropometric health indicator OR body composition assessment OR risk OR criteria OR classification OR health OR BIA OR bioelectrical impedance analysis OR dual-energy X-ray absorptiometry OR DXA OR ethnicity OR TC OR human shape OR MRI OR 3D) AND (Body mass index OR waist circumference OR waist-to-hip ratio OR waist-to-height ratio OR body fat percentage OR conicity index OR sagittal abdominal diameter OR Abdominal Volume Index OR Visceral Adipose Tissue Area OR Fat Free Mass Index OR Fat Mass Index OR Primary Shape and Shape Tendency OR Trunk Fat OR Trunk Fat Percentage Body Shape Index OR Body Roundness Index OR Neck circumference).

We also applied the restrictions included in the inclusion and exclusion criteria (Table 2).

### Eligibility and Data Abstraction

We reviewed the references identified by the search method specified above. The titles and abstracts were screened for inclusion. Full-text records were collated for the articles decided to be included and were further screened. Additional references were identified by manually searching the bibliographies of these articles.

Eligibility assessment and data abstraction were performed independently in an unblinded standardized manner by two reviewers (PP and SM-H). Abstracted data included eligibility criteria and methodological quality. Disagreements between reviewers were resolved by consensus.

## Results

A total of 42 AHIs have been identified and categorized into four groups according to the possibility of estimation from 3D human shapes and the availability of classification criteria (Figure 1 and Table 1). This study deeply reviews the 17 AHIs included in category 1 (Table 1).

### Literature Selection

**Figure 2:**
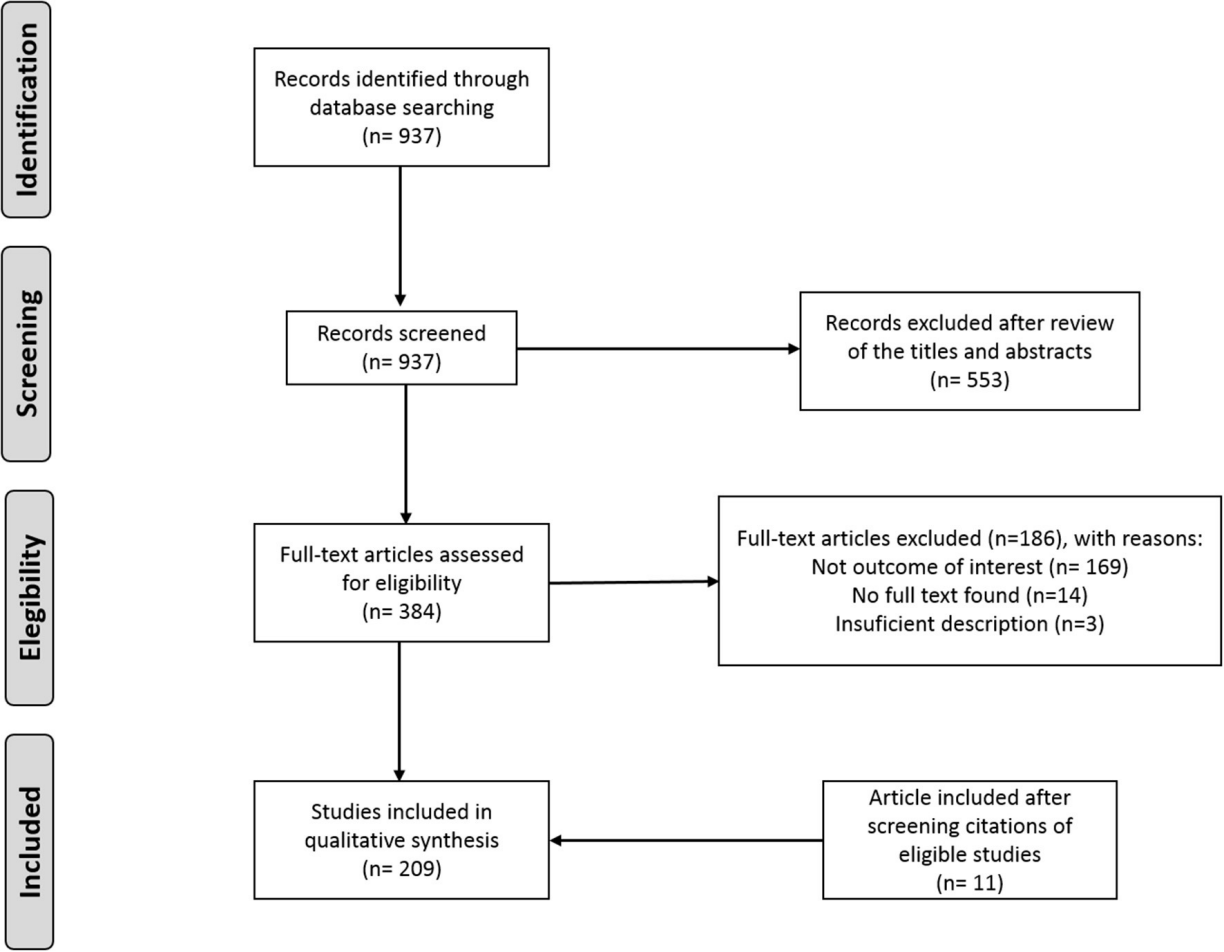
Flow diagram of the results from the literature search and the study selection.

The literature search in PubMed identified 937 records. All of them were screened. Finally, 209 of these records were considered eligible for inclusion in the current literature review. A detailed flow diagram of the results from the literature search and the study selection is shown in Figure 2.

### AHIs Selected for the Review

A list of the 17 AHIs reviewed is given in Table 1. The main body measurements used in the literature for their estimation are gathered in Table 3.

**Table 3:** Main parameters obtained from 3D human shapes and used to estimate AHIs.

**Body measurements**	**AHIs associated**
Weight	BMI, BFP, CI, VATA, FFMI, FMI, and ABSI
Height	BMI, WHtR, BFP, CI, VATA, FFMI, FMI, ABSI, and BRI
Waist circumference	WC, WHR, WHtR, BFP, CI, AVI, VATA, FFMI, FMI, TF, TFP, ABSI, and BRI
Hip circumference	WHR, BFP, and AVI
Thigh circumference	VATA and BFP
Volumes	BFP, FMI, PS&ST, and TF
Surface areas	BFP and PS&ST
Other body measurements	BFP, FFMI, FMI, PS&ST, and TF
Sagittal abdominal diameter	SAD
Neck circumference	NC

For each AHI, the main estimation or obtaining methods, health aspects associated, classification criteria, and limitations are described. The purpose of this paper is not to perform a comprehensive review of all published information, but to provide the most relevant data on each AHI.

### Body Mass Index

It is a simple and widespread indicator for the diagnosis of obesity. It is widely used as an index of relative weight.

#### Method

It is calculated as the ratio between body mass (in kilograms) and height squared (in meters).

$$\begin{array}{l} BMI=\frac{Body\;mass\;\left(kg\right)}{Height^{2}\left(m^{2}\right)} \end{array}$$

#### Associated Health Aspects

Both high and low BMIs are associated with an increased risk of developing chronic diseases and mortality. Different studies have shown a U-shaped association between BMI (higher than 25 kg/m^2^ and lower than 18.5–20 kg/m^2^) and all-cause mortality (Pischon et al., 2008; Song et al., 2015; Yi et al., 2015; Aune et al., 2016; Di Angelantonio et al., 2016). Furthermore, excess body weight has been associated with cancer burden (Lauby-Secretan et al., 2016; Sung et al., 2019; Hu et al., 2020). In the same line, evidence from several studies indicates that obesity or high BMI was associated with an increased risk of CVD events (Dwivedi et al., 2020) and an increased risk of diabetes (Ford et al., 1997; Resnick et al., 2000).

#### Classification Criteria

There are different cutoffs of BMI to classify individuals. BMI is considered normal when ranging from 18.5 to 24.9 kg/m^2^. Overweight is considered when BMI ranges from 25 to 29.9 kg/m^2^, and obesity when BMI is over 30 kg/m^2^. On the contrary, thinness is considered when BMI is under 18.5 kg/m^2^ (Yanovski and Yanovski, 2002; Bray et al., 2016). For children and adolescents, there have been developed reference-specific age and sex cutoffs to define child overweight and obesity (Rolland-Cachera et al., 1982; Deurenberg et al., 1991; Cole et al., 2000, 2007). In addition, in older subjects, the actual cutoffs to classify individuals should be modified. Furthermore, there are differences in body composition between populations of different ethnic origin. Because of that, different BMI cutoffs have been suggested for Asian population (WHO Expert Consultation, 2004; Wildman et al., 2004).

#### Limitations

BMI is the most widely used anthropometric index to estimate the overall body fatness. However, this index is a measure of excess weight rather than excess body fatness. The BMI has the advantage of simplicity (Romero-Corral et al., 2008), but it has deficiencies because it cannot directly address the aspects of body composition such as visceral fat or fat distribution (Ashwell et al., 2012; Böhm and Heitmann, 2013; Britton et al., 2013). Moreover, the BMI as an index for fat mass (FM) is therefore less reliable in the elderly because at an older age, there is a redistribution of body fat to the abdominal region (Seidell and Visscher, 2000). Furthermore, the morbidity associated with excess body weight varies among individuals of similar BMI and from different ethnicity (WHO Expert Consultation, 2004; Wildman et al., 2004). BMI is also inappropriate to classify subjects in the case of body builders or athletes because these subjects could be classified as obese. Finally, because BMI depends on age and height, adult population criteria should not be used to classify pre-pubertal and pubertal population (Rolland-Cachera et al., 1982). Gender should be also considered in these subjects.

### Waist Circumference

It is an easy and practical anthropometric index to evaluate the visceral fat in adults (Lemieux et al., 1996), which is well-correlated with the BFP (Heo et al., 2013).

#### Method

It is defined as the circumference at the waist; however, there is diversity in its measurement protocol, and there is currently no consensus (Ross et al., 2008). Some of the main leading health authorities consider the waist level to be located at the approximate midpoint between the lower margin of the last palpable rib and the top of the iliac crest (WHO, IDF, ISAK, ISO 7250, ASTM); or at the top of the iliac crest (NHANES III, NCEP ATP III); or at the narrowest waist (ASM); or at the level of the navel (MESA study).

#### Associated Health Aspects

Waist circumference is one of the main components of the metabolic syndrome (Expert Panel on Detection, Evaluation and Treatment of High Blood Cholesterol in Adults, 2001; Alberti et al., 2006). Increasing values of WC are an important cardiovascular risk factor, as well as are associated with other cardiovascular risk factors (Pouliot et al., 1994; Han et al., 1995; Rexrode et al., 1998; Dobbelsteyn et al., 2001). Raising of WC has also been related to type 2 diabetes risk (Chan et al., 1994; Carey et al., 1997; Wei et al., 1997). Finally, WC is strongly associated with all-cause and cardiovascular mortality (Pischon et al., 2008; Zhang et al., 2008; Cerhan et al., 2014; Song et al., 2015).

#### Classification Criteria

Diverse cutoffs have been proposed as criteria of metabolic syndrome. Recommended cutoffs (equal or higher) for WC vary for different ethnic groups and by gender (Balkau and Charles, 1999; Organization, 1999; Expert Panel on Detection, Evaluation and Treatment of High Blood Cholesterol in Adults, 2001; Alberti et al., 2006). The cutoffs for Europids are 94 cm for male and 80 cm for female. However, the cutoffs for central obesity adopted in the United States are 102 cm for male and 88 cm for female. Cutoffs recommended for South Asians, Chinese, and Japanese are 90 and 80 cm for men and women, respectively, which have been validated in a series of studies (Li et al., 2002; Lin et al., 2002; Zhou and Cooperative Meta-Analysis Group of the Working Group on Obesity in China, 2002; Tan et al., 2004; Wildman et al., 2004). For ethnic South and Central Americans, the cutoffs recommended are the South Asian recommendations until more specific data are available. Finally, for sub-Saharan Africans and for Eastern Mediterranean and Middle East individuals, the cutoffs recommended are the European recommendations until more specific data will be available (Alberti et al., 2006).

These criteria have also been modified for use in children and adolescents (Cook et al., 2003; Zimmet et al., 2007). The criteria established by the IDF specify cutoff points for the WC divided into the following age groups: 6–10, 10–16, and 16 years or more. It is suggested that below 16 years of age specific diagnostic criteria (higher than 90th percentile of the population) and above 16 years the IDF criteria for adults would be used (Zimmet et al., 2007).

#### Limitations

The procedure of waist measurement is not univocally standardized. Moreover, in subjects with BMI higher than 35 kg/m^2^, it is difficult to measure the WC. Also, WC is influenced by gender, age, and ethnicity. Finally, WC does not differentiate between visceral fat and subcutaneous fat.

### Waist-to-Hip Ratio

It is considered an indicator of visceral fat.

#### Method

It is defined as the quotient between the WC and the hip circumference (HC), in the same unit (dimensionless).

$$\begin{array}{l} WHR=\frac{WC\;}{HC\;} \end{array}$$

The WC can be measured by means of any of the above-mentioned protocols. HC is commonly measured at the level of the greatest projection at the back of the body (buttocks) (WHO, IDF, ISAK, ISO 7250, ASTM, NHANES III).

#### Associated Health Aspects

Waist-to-hip ratio has been associated with an increased risk of death, cardiovascular disease, and type 2 diabetes (Dalton et al., 2003; Motamed et al., 2015; Ross et al., 2020).

#### Classification Criteria

The World Health Organization recommended a WHR cutoff of 0.8 for women and 1 for men, as point used to show central obesity WHO Consultation on Obesity (1997).

#### Limitations

In many cases, ratios induce a loss of information and are not the most suitable to evaluate the visceral fat (van der Kooy et al., 1993). Furthermore, when BMI is higher than 35 kg/m^2^, there is more inaccuracy. WHR is also influenced by gender. Finally, there is not specific data for children.

### Waist-to-Height Ratio

It is a measure of body fat distribution. It is also called Index of Central Obesity (ICO).

#### Method

It is defined as the quotient between the WC and the height, in the same unit (dimensionless).

$$\begin{array}{l} WHtR=\frac{WC\;}{Height} \end{array}$$

#### Associated Health Aspects

WHtR is a good predictor of metabolic risk, even better than BMI and WC (Browning et al., 2010; Ashwell et al., 2012). WHtR has been associated with several chronic diseases (Ashwell et al., 2012, 2014). Higher values of WHtR have been associated with higher cardiometabolic risk (diabetes, hypertension, dyslipidemia, metabolic syndrome, and cardiovascular disease) (Ashwell et al., 2012; Han et al., 2017; Wang et al., 2018). Furthermore, WHtR has also been associated with cardiovascular and all-cause mortality (Schneider et al., 2010; Song et al., 2015).

#### Classification Criteria

The proposed cutoff value for WHtR as a marker of metabolic risk is 0.5 for different adult populations (Ashwell et al., 2012; Cai et al., 2013).

#### Limitations

There are no specific data for children.

### Body Fat Percentage

It is defined as the amount of FM as a percentage of the total body mass. The BFP is a core component of body composition, and it is strongly associated with obesity and the metabolic syndrome.

#### Method

There is a wide diversity of methods and models used to estimate it. However, there is not a universally recommended method to estimate BFP nor other important components of body composition (Wells and Fewtrell, 2006; Duren et al., 2008).

Body fat percentage can be estimated from body density, usually with Siri's and BroŽek's equations for adults (Siri, 1956; BroŽek et al., 1963). For children, there are equations similar to Siri's with age- and sex-specific constants (Lohman, 1989; Weststrate and Deurenberg, 1989; Wells et al., 2010).

Body density can be estimated with models based on skinfolds, such as age- and sex-specific equations for adults (Durnin and Womersley, 1974; Jackson and Pollock, 1978; Jackson et al., 1980), regression equations for adults involving as well WC (Lean et al., 1996), and regression equations for boys and girls of different maturation level (Deurenberg et al., 1990).

Body density can also be obtained by means of the following equation:

$$\begin{array}{l} Body\;density\;\left(kg/l\right)=\;\frac{Body\;mass\;\left(kg\right)}{Body\;volume\;\left(l\right)} \end{array}$$

where body volume can be obtained through magnetic resonance imaging (MRI); computed tomography (CT); underwater weighing (UWW); air displacement plethysmography (ADP); or through 3D scanners. Ng et al. (2016) correlated this latter body volume measurement with ADP volume and volume derived from DXA output using the equations of Wilson et al. (2012). Bourgeois et al. (2017) found good correlations for adults between the total body volume measured by diverse optical devices and that obtained from ADP and derived from DXA.

Body fat percentage can also be estimated by means of anthropometric measurements. Slaughter et al. (1988) establish regression equations for the prediction of BFP from the sum of two skinfolds in children and youth. Paul Deurenberg et al. (1991) propose a formula relating BFP to BMI, age, and sex for adults over 15 years and children under 15 years. Lean et al. (1996) propose 12 regression equations for adults predicting BFP for each sex group considering different combinations of variables: WC, age, triceps skinfold thickness, BMI, and other anthropometric measurements. Gallagher et al. (2000) developed prediction equations of BFP based on BMI, age, sex, and ethnicity, based on data collected from adults of three ethnic groups (White, African American, and Asian). Taylor et al. (2002) predict BFP in children and adolescents aged 3–18 years at BMI cutoffs at each age equivalent to overweight and obesity in adults. Wang et al. (2003) correlate BFP in adults of both sexes with WC measured at four commonly used sites. Bergman et al. (2011) propose a formula relating BFP to HC and height for adult men and women of different ethnicities, named by its authors body adiposity index (BAI). Gómez-Ambrosi et al. (2012) relate BFP to BMI, age, and sex by means of a formula for adults named Clínica Universidad de Navarra-Body Adiposity Estimator (CUN-BAE) by its authors. Lee et al. (2017) propose prediction equations of BFP for adults based on age, race, height, weight, circumference, and skinfold measures. Woolcott and Bergman (2018) propose an estimator of BFP for adults, named relative fat mass (RFM) based on height, WC, and sex. The reference method used in these studies for BFP estimation was DXA. Recently, Harty et al. (2020) used a rearrangement of the four-component model of Wang et al. (2002) as a reference to propose a new formula for predicting BFP from a set body metrics derived from an optical scanner (body surface area, and upper arm, calf, thigh, and upper and lower abdomen circumferences) and machine learning. This new formula is presented in two branches, one for leaner subjects and another one for larger subjects, to provide a better prediction for the leaner group.

Body fat percentage has also been derived from the whole-body silhouettes obtained from DXA scans in children aged 6–16 years (Xie et al., 2015).

Body fat percentage has also been correlated with 3D body shapes (shape descriptors), using perceptual-level geometry features to increase the prediction accuracy of regression models based on descriptors derived from direct 3D geometry, such as circumferences, surface areas, or volumes (Lu et al., 2018).

Also, Carletti et al. (2018) have investigated the possibility of performing image-based BFP estimation associated with DXA with depth images of fit male subjects, obtaining reasonably good accuracy.

#### Associated Health Aspects

High BFP has been associated with a high prevalence of cardiometabolic dysregulation, metabolic syndrome, type 2 diabetes, and cardiovascular risk factors (Romero-Corral et al., 2008; Gómez-Ambrosi et al., 2011; Shea et al., 2012; Kim et al., 2013; Ramírez-Vélez et al., 2017; Jia et al., 2018; Park et al., 2018; Escobedo-de la Peña et al., 2020). BFP has also been associated with a reduced survival (Padwal et al., 2016) and an increased risk of cardiovascular disease (Dervaux et al., 2008; Si et al., 2020) and cancer (Si et al., 2020).

#### Classification Criteria

Body fat percentage differs across populations (Deurenberg and Deurenberg-Yap, 2003). The BFP cutoffs for obesity in adult Caucasians proposed by the WHO are 25% for men and 35% for women, corresponding to a BMI of 30 kg/m^2^ (WHO | WHO | Physical status: the use and interpretation of anthropometry, 2020). There are also reports of BFP ranges for different ethnic groups: White, African American, and Asian (Gallagher et al., 2000). In the Chinese adult population, BFP cutoffs for obesity and for predicting the risk of cardiometabolic abnormalities have been proposed as 24% for men and 33% for women (Jia et al., 2018). There are also reference percentiles for children and adolescents according to age, gender (Taylor et al., 2002), and race (Mueller et al., 2004).

#### Limitations

Body fat percentage is age-, gender-, and ethnic-dependent (Deurenberg and Deurenberg-Yap, 2003). The current definitions of obesity using BFP are based on Western populations and probably need to be modified for Asian population.

### Conicity Index

It is a simple anthropometric index of body fat distribution to assess the central obesity with high accuracy (Valdez et al., 1993; Roriz et al., 2014).

#### Method

Conicity index has been defined as Valdez (1991):

$$\begin{array}{l} Conicity\;Index\;\left(CI\right)=\frac{WC\;\left(m\right)}{0,109\sqrt{\frac{Weight\;\left(kg\right)}{Height\;\left(m\right)}}} \end{array}$$

#### Associated Health Aspects

Conicity index has been associated with an excellent predictive capacity of metabolic syndrome in adolescents (Cristine Silva et al., 2020) and in older people (Ceolin et al., 2019). Furthermore, CI has demonstrated to be a good predictor of future diabetes (Andrade et al., 2016; Wang et al., 2019; Hernández-Vásquez et al., 2020) and hypertension (Andrade et al., 2016; Hernández-Vásquez et al., 2020). Thus, CI can be considered a good indicator of high cardiovascular risk (Tonding et al., 2014; Motamed et al., 2015), although it was not associated with coronary heart disease incidence in the population from the Framingham Heart Study (Kim et al., 2000).

#### Classification Criteria

Some cutoffs have been described in different populations (Almeida et al., 2009; Gadelha et al., 2016; Motamed et al., 2017; Filgueiras et al., 2019).

#### Limitations

There is a lack of specific cutoffs for clinical practice.

### Sagittal Abdominal Diameter

It is also referred to as abdominal height, which is well-correlated with abdominal visceral adipose tissue accumulation (Kvist et al., 1988; Kahn and Williamson, 1993; van der Kooy et al., 1993; Pouliot et al., 1994; Zamboni et al., 1998; Clasey et al., 1999), which is even better than WC and BMI (Yim et al., 2010).

#### Method

It measures the anteroposterior diameter of the abdomen, usually in the supine position. It can be measured with an abdominal caliper (de Souza and de Oliveira, 2013; Firouzi et al., 2018) or by diagnostic imaging techniques.

#### Associated Health Aspects

Sagittal abdominal diameter has been associated with components of the metabolic syndrome (Mukuddem-Petersen et al., 2006; Guzzaloni et al., 2009) and insulin resistance (Risérus et al., 2004; Petersson et al., 2007), which is a good predictor of glucose dysregulation and diabetes incidence (Gletsu-Miller et al., 2013; Pajunen et al., 2013; Kahn et al., 2014; Firouzi et al., 2018). Furthermore, SAD is strongly associated with cardiovascular risk (Richelsen and Pedersen, 1995; Kahn et al., 1996a; Gustat et al., 2000; Ohrvall et al., 2000; Rådholm et al., 2017) and increased mortality (Seidell et al., 1994; Kahn et al., 1996b; Empana et al., 2004; Iribarren et al., 2006). Finally, SAD has also been associated with the incidence of dementia (Whitmer et al., 2008).

#### Classification Criteria

There have been described cutoffs of SAD associated with elevated cardiometabolic risk in adults (Risérus et al., 2010). Furthermore, there are also cutoffs of SAD associated with the presence of obesity in children (Al-Daghri et al., 2010).

#### Limitations

Currently, one major obstacle for SAD utilization is the lack of widely acceptable reference ranges or cutoff values to assign to the risk categories of SAD (Vasques et al., 2015). Furthermore, the utilization of SAD requires a specific validation for each ethnicity.

### Abdominal Volume Index

It is an index that estimates the overall abdominal volume.

#### Method

This index is proposed by Guerrero-Romero and Rodríguez-Morán (2003) consisting of a formula that estimates the overall abdominal volume for adults through WC and HC:

$$\begin{array}{l} AVI=\frac{2{\left(WC\right)}^{2}+0.7{\left(WC-HC\right)}^{2}}{1,000} \end{array}$$

where WC is the minimum circumference at the umbilicus level, and HC is the maximum circumference at the symphysis of pubis level.

#### Associated Health Aspects

Abdominal volume index is a reliable and easy tool for the estimation of obesity (Guerrero-Romero and Rodríguez-Morán, 2003). Even it was suggested that it is a better index to assess the accumulation of fat in the abdominal area and is able to more accurately assess the BFP (Ehrampoush et al., 2017). AVI has been associated with metabolic syndrome in adolescents (Perona et al., 2019) and in adults (Motamed et al., 2017; Wang et al., 2017), with glucose dysregulation and diabetes (Guerrero-Romero and Rodríguez-Morán, 2003; Mamtani and Kulkarni, 2005) and with diabetes prediction (Wang et al., 2019). Also, AVI has been associated with cardiovascular risk (Motamed et al., 2015; Wang et al., 2018).

#### Classification Criteria

Although there are few data, cutoffs of AVI for the estimation of obesity have been described, suggesting that an AVI of ≥24.5 liters was associated with obesity (Guerrero-Romero and Rodríguez-Morán, 2003).

#### Limitations

There is a lack of specific cutoffs for clinical practice.

### Visceral Adipose Tissue Area

It is the surrounding area of adipose tissue in the visceral area, most commonly located at the level of the umbilicus (L4/5).

#### Method

Currently, CT and MRI are the gold standard methods for the quantitative evaluation of intra-abdominal adipose tissue (Shuster et al., 2012).

Brambilla et al. (2006) estimate VATA for adults with the WC by means of a regression formula. The model proposed by Samouda et al. (2013) correlates VATA with the WC and proximal thigh circumferences, BMI, and age, for adult men and women. Kuk et al. (2005) also obtained regression equations for adult men and women relating WC to VAT depending on sex and age.

#### Associated Health Aspects

Measurement of VATA is an accurate determination of adipose depot (Thaete et al., 1995). VATA has been associated with metabolic risk factors (Rosito et al., 2008). VATA has been associated with coronary stenosis and the presence and characteristics of coronary plaques (Ohashi et al., 2010; Kang et al., 2014).

#### Classification Criteria

There are not specific cutoffs for the general population. However, some cutoffs have been proposed. Various studies have defined VATA cutoffs associated with different components of the metabolic syndrome in Caucasians (Nicklas et al., 2003; Katzmarzyk et al., 2013), African American (Katzmarzyk et al., 2013), and Asian (Kim et al., 2006). In the same line, Seo et al. (2009) proposed the cutoffs for the presence of the metabolic syndrome.

#### Limitations

There is a lack of specific cutoffs for clinical practice.

### Fat-Free Mass Index

This index is associated with muscle mass.

#### Method

Fat-free mass index has been estimated by means of demographic information and components derived from the whole-body silhouettes obtained from DXA scans in children aged 6–16 years (Xie et al., 2015).

A good correlation of biomechanical variables with the FFMI was obtained by Campbell and Vallis (2014) in a stepwise regression. The resulting prediction equation that represents the largest FFMI variability in the study included forearm circumference (FC), BMI, maximum grip strength (MG), and the standard deviation of the double support time (SDDST) as predictor variables.

The FFMI is also obtained as the quotient between fat-free body mass (FFM) (in kilograms) and height squared (in meters).

$$\begin{array}{l} FFMI\left(\frac{kg}{m^{2}}\right)=\frac{FFM\;\left(kg\right)}{Height^{2}\;\left(m^{2}\;\right)} \end{array}$$

Lee et al. (2017) proposed the anthropometric prediction equations of FFM for adults based on age, race, height, weight, circumference, and skinfold measurements.

By definition, FFM can also be estimated from the BFP:

$$\begin{array}{l} FFM\left(kg\right)=Body\;mass\left(1-\frac{BFP}{100}\right) \end{array}$$

Fat-free mass can also be estimated by subtracting the FM from the total body mass.

$$\begin{array}{l} FFM=Body\;mass-FM \end{array}$$

This way, some prediction equations described in the next section have also been used to estimate FFM (Wang et al., 2003; Ng et al., 2016).

#### Associated Health Aspects

Fat-free mass index has been described in children, adults, and elderly subjects, as an indicator of nutritional status (VanItallie et al., 1990; Barlett et al., 1991; Schutz et al., 2002). Moreover, low FFMI has been associated with a higher risk of mortality in the general population (Zhu et al., 2003; Sørensen et al., 2020) and in older men (Graf et al., 2015). Also, FFMI had a strong positive relationship with blood pressure in children (Zhang and Wang, 2011).

#### Classification Criteria

There have been described percentiles of FFMI in Caucasians for age and gender (Schutz et al., 2002) and predicted FFMI by gender and ethnicity (Caucasian, African American, Hispanic, and Asian), according to age groups (Hull et al., 2011; Lu et al., 2012; Jin et al., 2019). Furthermore, percentiles of FFMI for the U.S. population aged 25–80 years, stratified by sex and independent of race-ethnicity, have been described (Kudsk et al., 2017). Also, age-based reference values for FFMI have been described for healthy children and adolescents from the United States (Shypailo and Wong, 2020) and adolescents from Spain (Durá-Travé et al., 2020).

#### Limitations

Fat-free mass index is age-, gender-, and ethnic-dependent. Furthermore, more data are necessary for children.

### Fat Mass Index

This index is associated with FM.

#### Method

The FMI is calculated as the quotient between FM (in kilograms) and height squared (in meters).

$$\begin{array}{l} FMI\left(\frac{kg}{m^{2}}\right)=\frac{FM\;\left(kg\right)}{Height^{2}\;\left(m^{2}\right)} \end{array}$$

Wang et al. (2003) correlate WC measured at four commonly used sites with body FM in adults of both sexes. Ng et al. (2016) propose a model to estimate FM from the WC, the waist width, the average leg volume, sex, and the torso volume obtained from 3D scan measurements. FM has also been correlated with the central obesity depth, thigh volume, sex, and torso volume measured by stereovision body imaging (Lee et al., 2015). Farina et al. (2016) establish sex-specific regression models to predict FM from height, weight, and parameters obtained from a single 2D side view photo taken with a smartphone, with promising results compared to DXA measurements. Lee et al. (2017) propose the prediction equations of FM for adults based on age, race, height, weight, circumference, and skinfold measurements.

Fat mass index has also been estimated by means of demographic information and components derived from the whole-body silhouettes obtained from DXA scans in children aged 6–16 years (Xie et al., 2015).

By definition, FM can also be estimated from the BFP:

$$\begin{array}{l} FM\left(kg\right)=Body\;mass\left(\frac{BFP}{100}\right) \end{array}$$

Fat mass index can also be calculated as:

$$\begin{array}{l} FMI\left(\frac{kg}{m^{2}}\right)=BMI-FFMI \end{array}$$

#### Associated Health Aspects

Fat mass index accurately classifies obesity (Peltz et al., 2010). Also, FMI seems to be a better screening tool in the prediction of the presence of metabolic syndrome than BMI and BFP in men and women (Liu et al., 2013). Furthermore, high FMI has been associated with a higher risk of mortality in the general population (Sørensen et al., 2020), although other studies did not show the impact of FMI on mortality (Graf et al., 2015).

#### Classification Criteria

There have been described percentiles of FMI in Caucasians for age and gender (Schutz et al., 2002). Reference intervals for FMI in Chinese adults have also been proposed for a given age and gender category (Lu et al., 2012) and by ethnicity (Jin et al., 2019). Finally, age-based reference values for FMI have been described for healthy children and adolescents from the United States (Shypailo and Wong, 2020) and adolescents from Spain (Durá-Travé et al., 2020).

#### Limitations

Fat mass index is age-, gender-, and ethnic-dependent. More data are necessary for children and adults.

### Primary Shape and Shape Tendency

Ratios between torso height, area, and volume that allow to estimate the type of torso (fat) volume distribution in individuals with morbid obesity, and its “intensity” or “degree.”

#### Method

The indices are proposed by Stefan et al. (2014) and Stefan and Gilbert (2015) and are based on measurements (linear and circumferential measurements, volume, and surface data) obtained from 3D scans of torso segments in morbidly obese individuals.

$$\begin{array}{l} Torso\;Volume/Surface\;area\;\left(TVSA\right)\;ratio\\ \;=\frac{Torso\;Volume}{Torso\;Surface\;Area}\\ \;BariPlex\;=\frac{Torso\;Height}{TVSA\;ratio}\\ Superior\;Torso\;BariPlex\;=\;\frac{Superior\;Torso\;Height}{Superior\;TVSA\;Ratio}\\ \;Inferior\;Torso\;BariPlex\;=\;BariPlex-Superior\;Torso\;BariPlex\\ \;Primary\;Shape=\frac{Superior\;Torso\;BariPlex\;}{BariPlex\;}\\ \;Shape\;Tendency=\frac{Inferior\;Torso\;BariPlex}{Superior\;Torso\;BariPlex} \end{array}$$

#### Associated Health Aspects

Primary shape allows us to identify and classify the individuals according to the obesity phenotype based on the fat distribution as “android” or “gynecoid.” Android phenotype has been associated with a worse metabolic profile. It has been shown that these “shape descriptors” remain consistent throughout the weight loss. Thus, both can be used to document the measurements of preoperative morbidly obese individuals and to track their measurement changes periodically after weight loss surgery as part of the postoperative follow-up visits (Stefan and Gilbert, 2015).

#### Classification Criteria

A scale has been described for PS indicator for determining morbidly obese body fat distribution as gynecoid (≥0.8), mixed (0.71–0.79), or android (≤0.7). A scale has also been described for ST, which informs about the degree of the PS (Stefan and Gilbert, 2015).

#### Limitations

These indicators have been only evaluated in subjects with morbid obesity.

### Trunk Fat

It is a measure of the amount of FM in the trunk (in kilograms).

#### Method

Trunk fat has been well-correlated with WC in children and adolescents (Taylor et al., 2000). TF has been estimated from seven skinfold measurements: triceps, biceps, chest, subscapular abdomen, thigh, and calf (He et al., 2002). Wang et al. (2003) correlated TF with WC measured at four commonly used sites in adults of both sexes. TF has also been correlated with the central obesity depth, sex, and torso volume measured by stereovision body imaging (Lee et al., 2015). Ng et al. (2016) propose a model to estimate TF in healthy adults by means of the WC, the waist width, sex, and torso volume.

Recently, Lu and Hahn (2019) have proposed a model to extrapolate 3D voxel-level body composition using multimodality registration based on 3D body shape derived from an optical body scan system, 2D pixel-level body composition reference derived from DXA, and a generic body composition template with anatomically accurate human skin, muscle, and skeleton system. This model shows promising results for TF distribution.

#### Associated Health Aspects

Trunk fat is a predictor of blood pressure in African American, Asian, and Caucasian boys at all stages of puberty (He et al., 2002). Greater TF has also been associated with insulin resistance (Grunfeld et al., 2007), higher glucose levels (Snijder et al., 2004), and components of the metabolic syndrome (Wiklund et al., 2008).

#### Classification Criteria

Cutoffs by age and gender for identifying high TF have been proposed in children and adolescents (Taylor et al., 2000).

#### Limitations

There are no data for adult population.

### Trunk Fat percentage

It is the percentage of FM in the trunk with respect to the total trunk mass.

#### Method

It is defined as the quotient between the FM in the trunk area (TF) and the total trunk mass (in the same unit).

$$\begin{array}{l} TFP=\frac{TF}{Trunk\;mass}100 \end{array}$$

Wang et al. (2003) correlated TFP with WC measured at four commonly used sites in adults of both sexes.

#### Associated Health Aspects

Trunk fat percentage is obtained from TF. Thus, we can assume that it is well-correlated with WC (Taylor et al., 2000), blood pressure (He et al., 2002), insulin resistance (Grunfeld et al., 2007), glucose levels (Snijder et al., 2004), and components of the metabolic syndrome (Wiklund et al., 2008).

#### Classification Criteria

Cutoffs for TFP have been proposed for high risk of inflammatory markers in children (Karatzi et al., 2016). Cutoffs for elevated risk of insulin resistance in children have also been described (Moschonis et al., 2016).

#### Limitations

There are no data for adult population.

### A Body Shape Index

It reports on body shape and concentration of body volume.

#### Method

It is an index proposed by Krakauer and Krakauer (2012) based on WC adjusted for height and weight, defined as the WC divided by the BMI raised to two-thirds and by the square root of the height.

$$\begin{array}{l} ABSI=\frac{WC}{BMI^{2/3}\;\cdot Height^{1/2}} \end{array}$$

#### Associated Health Aspects

A body shape index has been shown to be associated with adipose abdominal tissue (Krakauer and Krakauer, 2012), with components of the metabolic syndrome (Bertoli et al., 2017), cardiometabolic risk (Cheung, 2014; Fujita et al., 2015; Wang et al., 2018), and the presence (Chang et al., 2015) or onset of diabetes (He and Chen, 2013; Fujita et al., 2015; Han et al., 2017). Furthermore, ABSI was associated with carotid atherosclerosis (Geraci et al., 2019), stroke incidence in men (Abete et al., 2015), and an increased risk of CVD incident in the adult population (Bozorgmanesh et al., 2016). Finally, ABSI predicted mortality from CVD (Song et al., 2013, 2015) and was also a robust predictor of all-cause mortality (Krakauer and Krakauer, 2012; Song et al., 2015; Grant et al., 2017; Sato et al., 2017; Lee et al., 2018).

#### Classification Criteria

Mean values of ABSI have been described by age and gender in a large adult population (Krakauer and Krakauer, 2012).

#### Limitations

A body shape index depends on age and gender. There are no data for children. Furthermore, there are not specific cutoffs for clinical practice.

### Body Roundness Index

An index developed by Thomas et al. (2013) attempts to quantify the individual body shape in a height-independent manner.

#### Method

It is a formula based on the WC and height.

$$\begin{array}{l} \;BRI=364.2-365.5\;\cdot Eccentricity\\ Eccentricity=\sqrt{1-\frac{1}{\pi^{2}}{\left(\frac{WC\;\left(m\right)}{Height\;\left(m\right)}\right)}^{2}} \end{array}$$

#### Associated Health Aspects

Body roundness index was useful to predict the insulin resistance (Feng et al., 2019) and has a good discriminatory power for metabolic syndrome in adults from diverse populations (Li et al., 2019; Rico-Martín et al., 2020). BRI was also associated with the presence of diabetes mellitus (Chang et al., 2015) and higher cardiovascular risk (Maessen et al., 2014; Wang et al., 2018).

#### Classification Criteria

Cutoffs of BRI have been proposed in adults at high risk of cardiometabolic abnormalities (Tian et al., 2016).

#### Limitations

There is a lack of specific cutoffs for clinical practice. Furthermore, the complexity for the calculation of BRI compared to the simplicity of other indicators, such as BMI or WC, makes difficult the generalization of its use.

### Neck Circumference

It is a relatively new, inexpensive, and practical measure to identify obesity (Ben-Noun et al., 2001; Hingorjo et al., 2012; Aswathappa et al., 2013). It is a marker of upper body subcutaneous adipose tissue distribution (Aswathappa et al., 2013).

#### Method

It is measured at the point just below the bulge at the thyroid cartilage (Adam's apple).

#### Associated Health Aspects

The NC is a simple measure that could be used to identify obesity in both adults and children (Ben-Noun et al., 2001; Hingorjo et al., 2012; Lou et al., 2012). The NC is also associated with other anthropometric measures for obesity and body distribution (Preis et al., 2010). Furthermore, some studies have indicated that NC could be considered as an independent correlate of cardiometabolic risk factors (Ben-Noun and Laor, 2003, 2004, 2006; Preis et al., 2010; Guo et al., 2012).

#### Classification Criteria

Different cutoffs for NC have been proposed to identify subjects with overweight/obesity (Ben-Noun et al., 2001; Hingorjo et al., 2012). However, there are not enough data to establish the utility of these cutoffs.

#### Limitations

There is a lack of specific cutoffs for clinical practice.

## Discussion

Obesity is one of the most prevalent metabolic disorders worldwide, which is a known risk factor for non-communicable diseases: metabolic syndrome, type 2 diabetes, CV disease, arthrosis, cancer, and depression. Obesity has a high impact on metabolic disturbances, contributing to the development of insulin resistance, atherogenic dyslipidemia, metabolic syndrome, and non-alcoholic fatty liver disease, leading to the development of type 2 diabetes and cardiovascular disease. However, not all obese subjects develop metabolic complications (Smith et al., 2019). Therefore, it is important to detect those most at risk. In this sense, the AHI have become a fundamental tool for the identification, intervention, or evaluation of the impact of risks. This study identified and analyzed 17 AHIs that can be obtained or estimated from 3D human shapes.

Among the 17 AHIs, the main ones used in the literature for their estimation are shown in Table 1. Measuring tapes have traditionally been used for circumferences and lengths. For volume measurements, air and water displacement techniques have been commonly used to estimate the total body volume. Other techniques such as CT or MRI are useful to assess the total and regional body volume. However, all of these techniques are invasive in one way or another, require well-trained laboratory personnel, and are far too expensive for field settings, making obtaining AHI not very accessible for an extensive diagnosis of obesity and other health risk factors.

In this sense, studies that use traditional techniques have been complemented or replaced by studies that use 3D imaging devices and other emerging, inexpensive, and accessible technologies that make obtaining body dimensions easy and reliable (Medina-Inojosa et al., 2016; Ballester et al., 2018). The body measurements obtained with these new technologies have been correlated with reference methods (Ng et al., 2016; Bourgeois et al., 2017; Tinsley et al., 2020). In addition, these novel technologies have opened a way to evaluate other body parameters such as surface areas or AHIs shape descriptors (Stefan et al., 2014; Lu et al., 2018), whose application has even been improved by machine learning techniques (Harty et al., 2020).

From the 17 AHIs evaluated, those with the most published references on the subject covered by our review were BMI, WC, BFP, VATA, SAD, FFMI, FMI, ABSI, and BRI. However, until now, none of the AHIs is considered a sufficiently accurate method for the assessment of body composition in obesity. All of them have shown advantages and limitations. The AHIs most widely used in clinical practice are BMI and WC. In fact, the definition of obesity is based on BMI cutoffs. BMI is readily available, and there are the well-established cutoffs for the population. It is useful to estimate the overall body fat; however, it is not appropriate to evaluate the visceral fat or fat distribution (Ashwell et al., 2012; Böhm and Heitmann, 2013; Britton et al., 2013). Other AHIs such as WC or those that include waist measurement are well-correlated with visceral fat (Lemieux et al., 1996; Ashwell et al., 2012) being also used routinely in clinical practice. However, these are also imperfect as indicators of intra-abdominal fat because they also include subcutaneous fat deposition. On the contrary, there are AHIs that provide more information on the distribution of body fat, but it is necessary to use complex, invasive, and/or expensive techniques (BIA, DXA, CT, etc.) to obtain them (Duren et al., 2008). Therefore, they are unsuitable for clinical practice. On the other hand, in addition to the inherent limitations of any individual AHI, only BMI, WC, and WHR offer the well-established cutoff values for classifying patients. In fact, the cutoff values for the rest of AHIs are not yet validated. Furthermore, universal cutoffs may not be applicable to certain ethnic groups. In recent years, interest has focused on estimating or predicting these AHIs from 3D scanning systems through a novel set of measurements (Giachetti et al., 2015), integrating perceptual-level geometry characteristics to regression equations (Lu et al., 2018), using multimodal registration (Lu and Hahn, 2019), or statistical modeling of shapes (Ng et al., 2019), and in the evaluation of the validity of the AHIs using reference methods (Ryder, 2010; Adler et al., 2017; Harbin et al., 2018; Wong et al., 2019; Tinsley et al., 2020). 3D scanning systems could easily help identify the regional distribution of fat, which in fact critically determines the overall effects of obesity on health risks.

The studies reviewed have demonstrated the potential of 3D imaging techniques and other emerging technologies to estimate AHIs, particularly those related to body composition, which are now on the rise due to their usefulness. However, they reflect that the predictive equations of body composition still need to be refined to be considered an accurate method. Additionally, only a few AHIs have widely accepted cutoffs that can be used routinely to assist the clinician. Finally, the present study has some limitations. Interesting articles may be missing from the scope of our review because we have analyzed articles found in PubMed but not manuscripts included in other databases. In addition, we have carried out the bibliographic search with a limiting title and/or abstract field. Future work may also target AHIs of Categories 2, 3, and 4 for 3D body shape estimates or extended classification criteria.

## Conclusion

This review provides an overview of all AHIs, with particular emphasis on those that can be obtained or estimated from 3D human shapes, due to the rise and potential of 3D scanning systems. 3D scanning systems and other emerging technologies can play an important role in the prevention and early detection of obesity and other health risk factors, due, among other aspects, to their accessibility, and can even be used in primary care.

Our findings reveal that although many AHIs have emerged, until now there is no single method sufficiently adequate for the estimation of adiposity and its distribution and, therefore, for the evaluation of obesity and other risk factors for health. Future studies are still needed to improve body composition prediction methods, with widely accepted cutoff points for clinical practice, and for the study of AHIs of Categories 2, 3, and 4 to convert them into Category 1.

## Author Contributions

PP, JD-G, AB, and SM-H conceptualized and structured the manuscript, performed the general search, review, selection, and classification of the references. PP and SM-H wrote the manuscript. PP, SM-H, AB, JRea, and JRed participated in the manuscript editing and revised it for important intellectual content. All authors have reviewed and approved the final manuscript.

## Conflict of Interest

The authors declare that the research was conducted in the absence of any commercial or financial relationships that could be construed as a potential conflict of interest.
